# Supplementary material for: Development and characterization of phospho-ubiquitin antibodies to monitor PINK1-PRKN signaling in cells and tissue
Source: Autophagy. 2024 May 27;20(9):2076–91. doi: 10.1080/15548627.2024.2356490 (PMC11346534; doi:10.1080/15548627.2024.2356490)
Supplement: Supplementary Material_Watzlawik and Hou_revision R2.docx [file KAUP_A_2356490_SM9996.docx]

# SUPPLEMENTARY MATERIAL

# Development and characterization of phospho-ubiquitin antibodies to monitor PINK1-PRKN signaling in cells and tissue

Jens O. Watzlawik^1,#^, Xu Hou^1,#^, Tyrique Richardson^1^, Szymon L. Lewicki^1^, Joanna Siuda^2^, Zbigniew K. Wszolek^3^, Casey N. Cook^1,4^**,** Leonard Petrucelli^1,4^, Michael DeTure^1^, Dennis W. Dickson^1,4^, Odetta Antico^5^, Miratul M. K. Muqit^5^, Jordan B. Fishman^6^, Karima Pirani^7^, Ravindran Kumaran^8^, Nicole K. Polinski^9^, Fabienne C. Fiesel^1,4^, and Wolfdieter Springer^1,4^ *

^1^ Department of Neuroscience, Mayo Clinic, Jacksonville, FL 32224, USA

^2^ Department of Neurology, Faculty of Medical Sciences in Katowice, Medical University of Silesia, Katowice 40-055, Poland

^3^ Department of Neurology, Mayo Clinic, Jacksonville, FL 32224, USA

^4^ Neuroscience PhD Program, Mayo Clinic Graduate School of Biomedical Sciences, Jacksonville, FL 32224, USA

^5^ MRC Protein Phosphorylation and Ubiquitylation Unit, School of Life Sciences, University of Dundee, Dundee, DD1 5EH, United Kingdom

^6^ 21st Century Biochemicals Inc., Marlborough, MA 01752, USA

^7^ ImmunoPrecise Antibodies Ltd., Victoria, BC V8Z 7X8, Canada

^8^ Abcam plc, Cambridge, CB2 0AX, United Kingdom

^9^ The Michael J. Fox Foundation for Parkinson’s Research, New York, NY 10163, USA

*^#^* Contributed equally

* Correspondence should be addressed to:

Wolfdieter Springer, PhD

Department of Neuroscience; Mayo Clinic

4500 San Pablo Road, Jacksonville, FL 32224, USA

E-mail: [Springer.Wolfdieter@mayo.edu](mailto:springer.wolfdieter@mayo.edu)

Tel: +1 904 953 6129; Fax: +1 904 953 7117

**Running title:** Development of recombinant phospho-ubiquitin antibodies


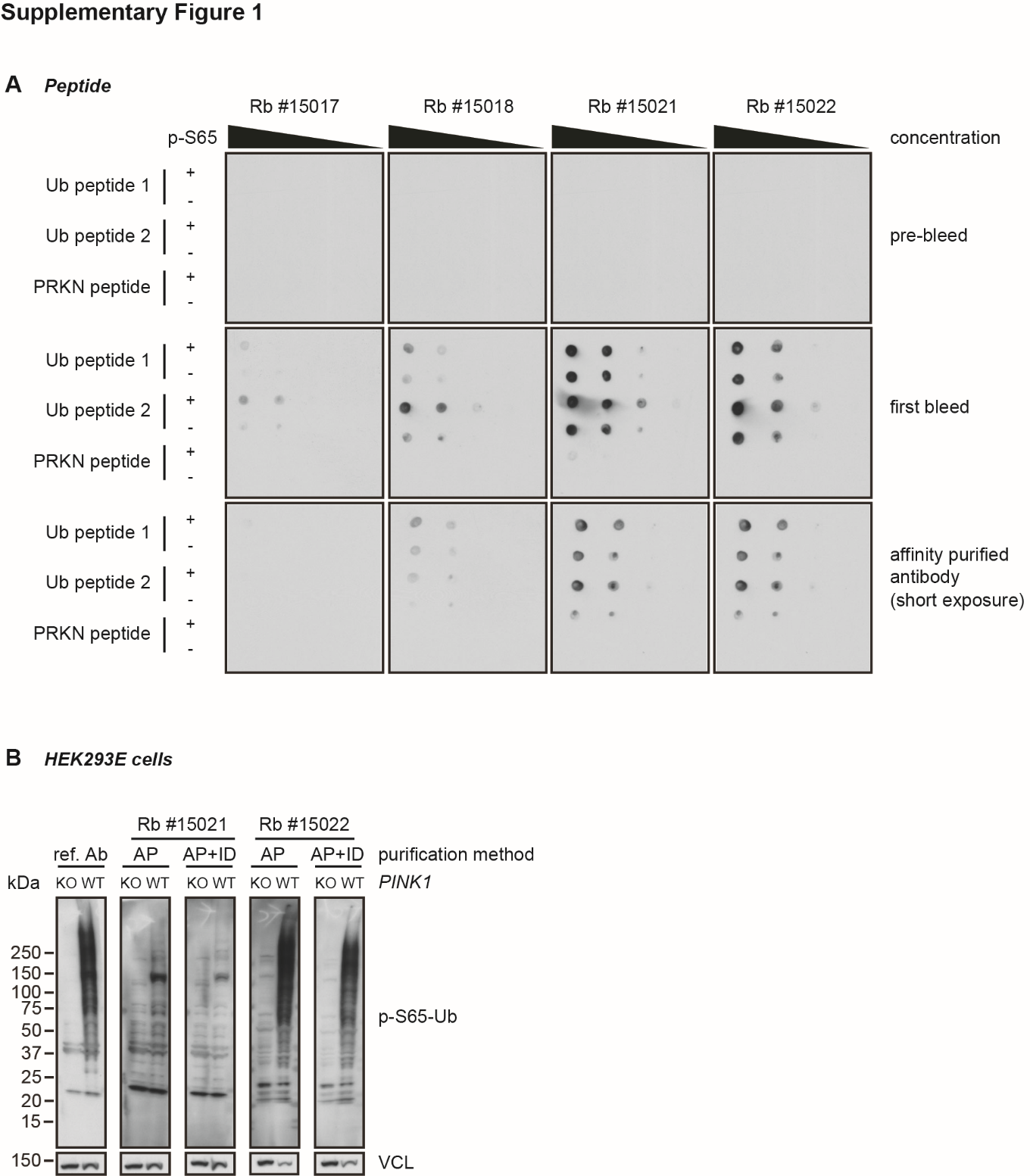


**Figure S1.** Identification of p-S65-Ub immunopositive bleeds in four rabbits. (**A**) Dot blot analyses to identify p-S65-Ub-positive bleeds from immunized rabbits. Two sets of 13-mer p-S65-Ub peptides containing the p-S65 in its center position that were used for rabbit immunizations as well as negative controls from their non-phosphorylated counterparts and 12-mer non-/phosphorylated PRKN peptides containing the S65 in its center position were spotted on membranes in different concentrations (0.2-25 µM; 5-fold serial dilution). Blots were probed with rabbit sera from before immunization (pre-bleed), after immunization (first bleed), or after affinity-purification. (**B**) WT and *PINK1* KO HEK293E cells were treated for 8 h with 20 µM CCCP and cell lysates were used for western blot analyses. Blots were probed with the reference antibody or bleeds from two rabbits (Rb #15021 and #15022) with either affinity purification (AP) alone or also immuno-depletion (AP+ID). VCL was used as loading control. AP: affinity purification; ID: immuno-depletion; KO: knockout; Rb: rabbit; Ref. Ab: reference antibody; WT: wild-type.

**
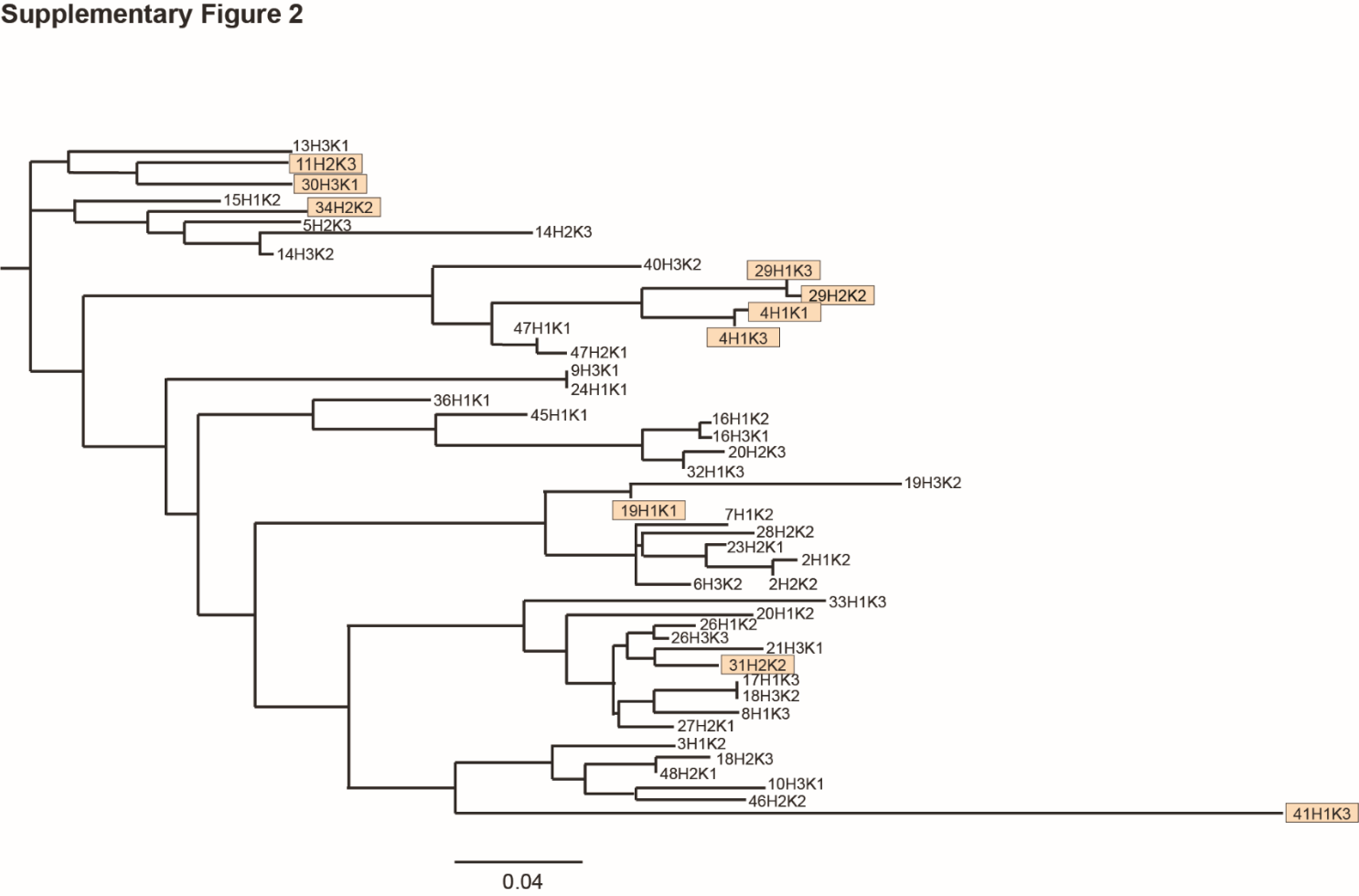
**

**Figure S2.** Phylogenetic sequence tree of p-S65-Ub clone variable region. A phylogenetic sequence tree was generated based on the p-S65-Ub antibody clone amino acid sequence for combined heavy and kappa variable regions. The top ten promising p-S65-Ub clone supernatants are highlighted in light orange. Scale bar: 0.04 nucleotide substitutions per site.

**
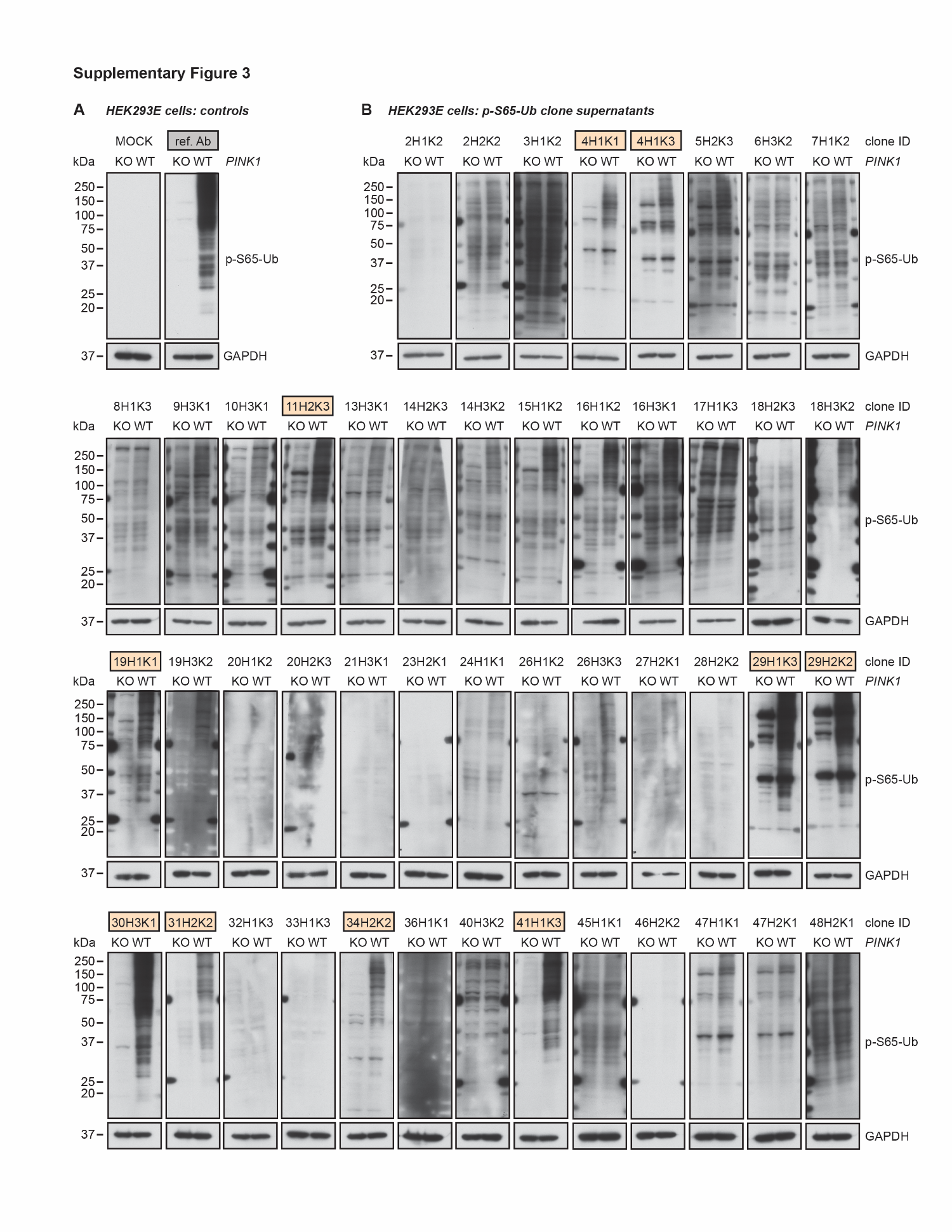
Figure S3.** Western blot screening of top recombinant p-S65-Ub clone supernatants. WT and *PINK1* KO HEK293E cells were treated for 24 h with 20 µM CCCP and cell lysates were used for western blot analyses. (**A**) Representative western blot images from supernatant produced by HEK293 cells transfected with a media control containing all media and transfection reagent except the plasmids (MOCK) vector that served as negative control or from the reference antibody that served as positive control. (**B**) Representative western blot images from 47 p-S65-Ub recombinant antibody supernatants. GAPDH was used as loading control. The top ten promising p-S65-Ub antibody clones are highlighted in light orange. ref. Ab: reference antibody; KO: knockout; WT: wild-type.

**
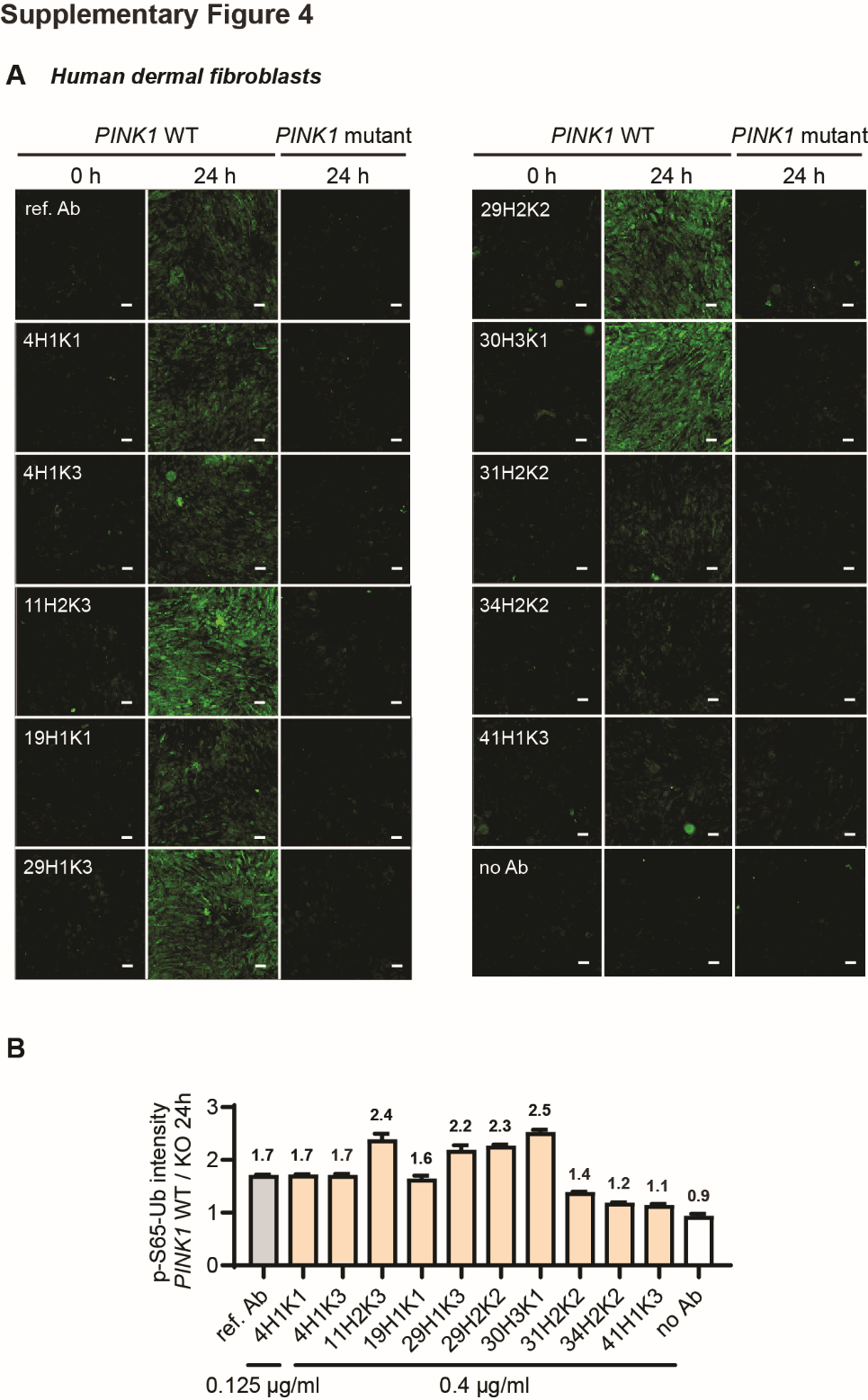
**

**Figure S4.** Characterization of the top recombinant p-S65-Ub clone supernatants for immunocytochemistry staining in human dermal fibroblasts. All ten p-S65-Ub clone supernatants (0.4 μg/ml) and the reference antibody (0.125 μg/ml) were evaluated by immunocytochemistry in fibroblasts treated with 2 μM valinomycin for 0 or 24 h. (**A**) Representative images of p-S65-Ub immunoreactive signals (green) in human primary skin fibroblasts carrying WT or homozygous *PINK1*^Q456X^ mutation. (**B**) Fluorescence intensities of each clone were quantified by high content imaging and compared relative to the treated *PINK1* mutant fibroblasts. Fold changes are labeled at the top of each bar. N = 2. Samples stained without primary antibody (no Ab) was used a negative control. Scale bar: 50 μm. ref. Ab: reference antibody; WT: wild-type.


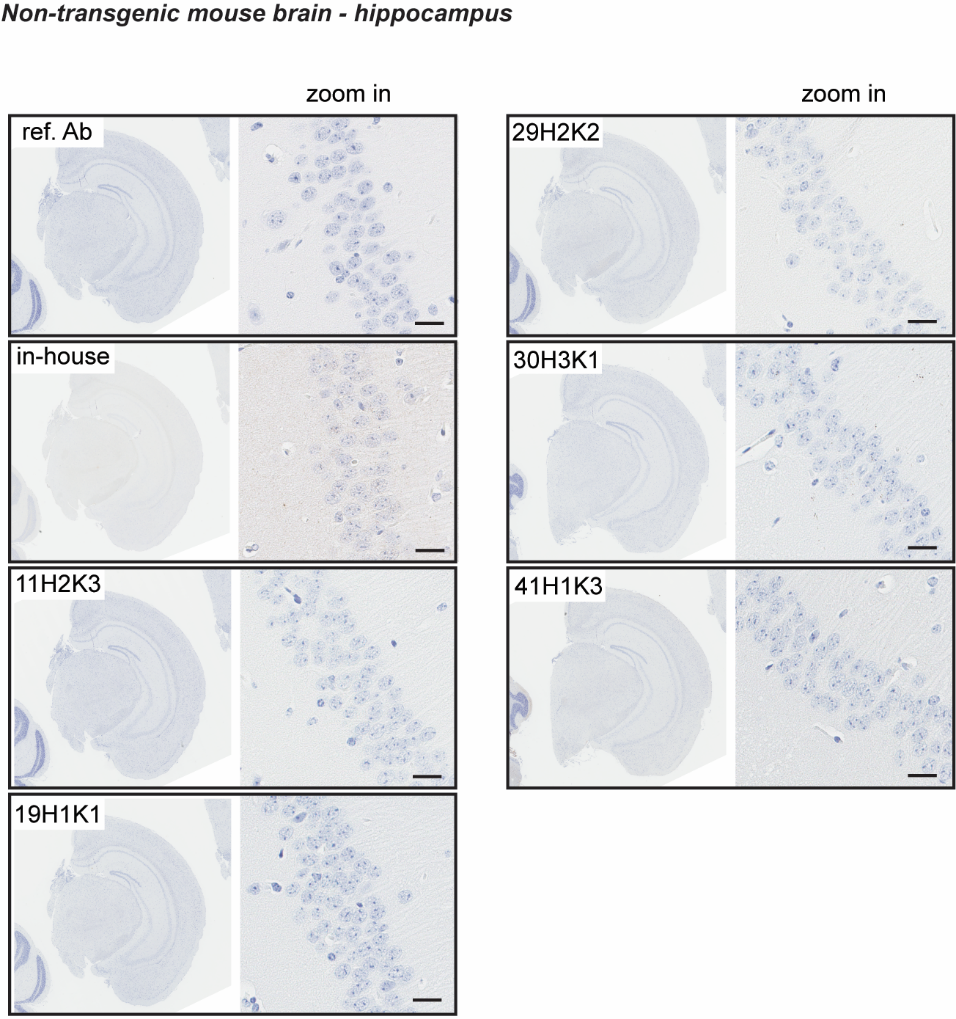


**Figure S5.** Characterization of the top p-S65-Ub antibodies by immunohistochemistry staining of mouse brain. All five p-S65-Ub recombinant antibodies together with our in-house p-S65-Ub antibody and the reference antibody were evaluated by immunohistochemistry in serial brain sections from two non-transgenic mice. Same concentration (1.56 μg/ml) was used for all antibodies. The entire region of the stained hemibrain is shown to the left at 1.5x magnification. Zoom-in images of hippocampal regions are shown to the right. No immunoreactive signal was observed in brains of non-transgenic mice. Scale bar: 50 μm. ref. Ab: reference antibody.
